# Harmane inhibits hepatic cytochrome P450 enzymes in rats

**DOI:** 10.3389/fphar.2026.1863858

**Published:** 2026-08-17

**Authors:** E. Klásková, O. Zendulka, O. Peš, S. M. Eyrilmez, D. Bednář, J. Juřica

**Affiliations:** 1 Department of Pharmacology, Faculty of Medicine, Masaryk University, Brno, Czechia; 2 Department of Biochemistry, Faculty of Medicine, Masaryk University, Brno, Czechia; 3 Loschmidt Laboratories, Department of Experimental Biology and RECETOX, Faculty of Science, Masaryk University, Brno, Czechia; 4 International Clinical Research Center, St. Anne’s University Hospital Brno, Brno, Czechia; 5 Hospital Pharmacy, Masaryk Memorial Cancer Institute, Brno, Czechia; 6 Department of Pharmacology and Toxicology, Faculty of Pharmacy, Masaryk University, Brno, Czechia

**Keywords:** cytochrome P450, drug interaction, harmane, inhibition, rat, ß-carboline

## Abstract

**Introduction:**

Harmane (HAR) is a beta‐carboline alkaloid found in plants, heat‐processed foods, and tobacco. It is an active component of widely used antidepressant and anxiolytic herbs. In this study, we investigate the effects of HAR on rat CYP enzymes both *in vivo* and *in vitro* and examine the interactions between HAR and human CYP enzymes through *in silico* methods.

**Methods:**

The impact of subchronic premedication with HAR at doses of 25, 40, and 64 mg/kg on CYP enzyme activity was evaluated in rats using CYP‐specific probe substrates of CYP1A2, CYP2A, CYP2B, CYP2C6, CYP2C11, CYP2D1/2, and CYP3A1/2. *In vitro* inhibitory assays of HAR were conducted. Finally, HAR was docked *in silico* into human CYP enzyme active sites.

**Results and Discussion:**

HAR premedication significantly decreased the protein content and metabolic activity of rat CYP2B1, CYP2C11, CYP2D1/2, and CYP3A1/2. *In vitro*, HAR showed moderate inhibition of CYP2A1 and CYP3A1/2 (IC50 values of 14.0 and 1.2 µM, respectively) and no inhibition of the other enzymes tested. *In silico* docking studies with human CYP enzymes confirmed that HAR has the potential for CYP‐mediated drug–drug inhibitory interactions. In summary, HAR significantly inhibited the activities of several drug‐metabolizing CYP enzymes in the preclinical model. These findings suggest the need for further research into HAR’s pharmacokinetic interactions.

## Graphical Abstract

**Figure:**
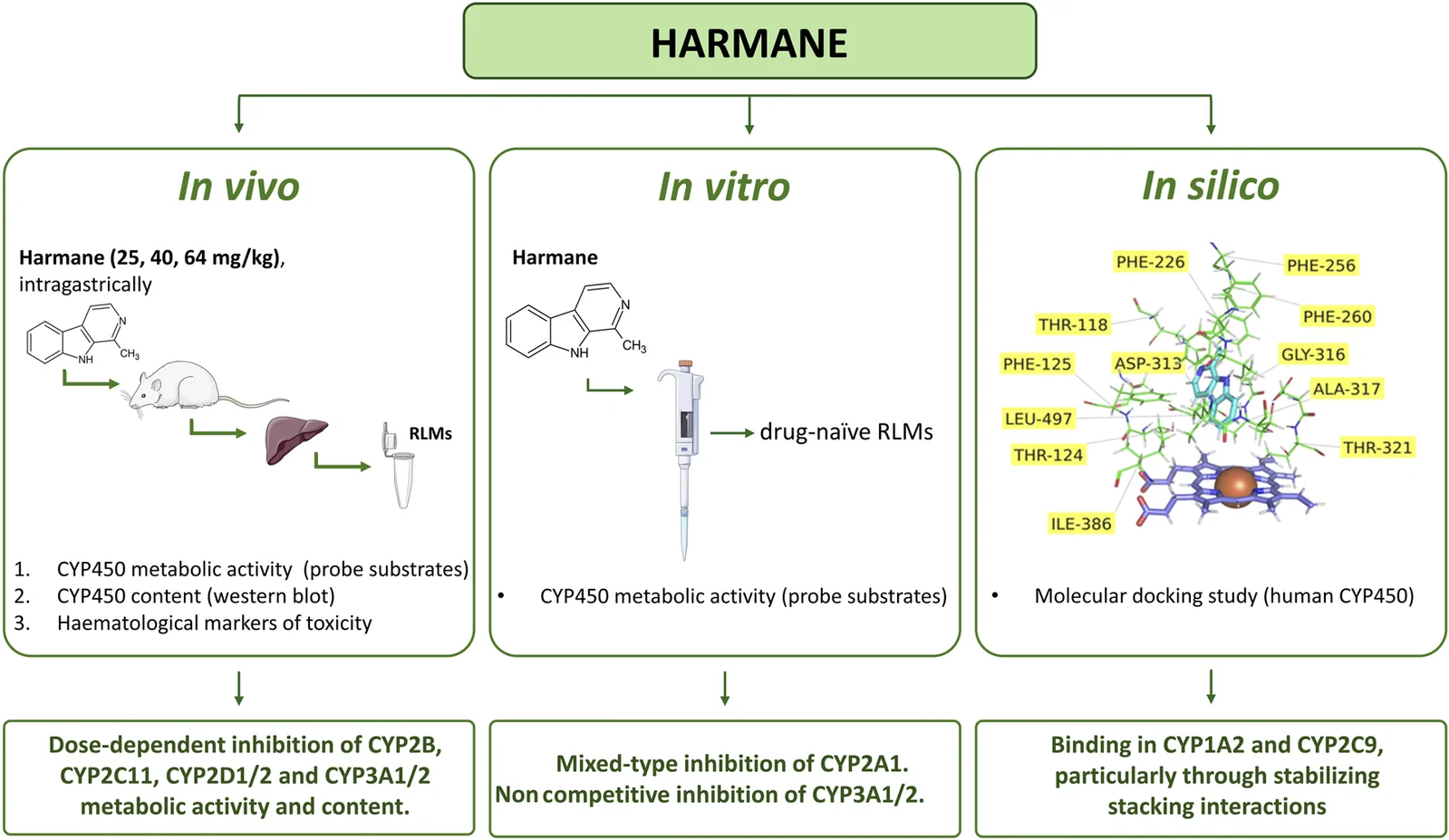

## 1 Introduction

**FIGURE 1:**
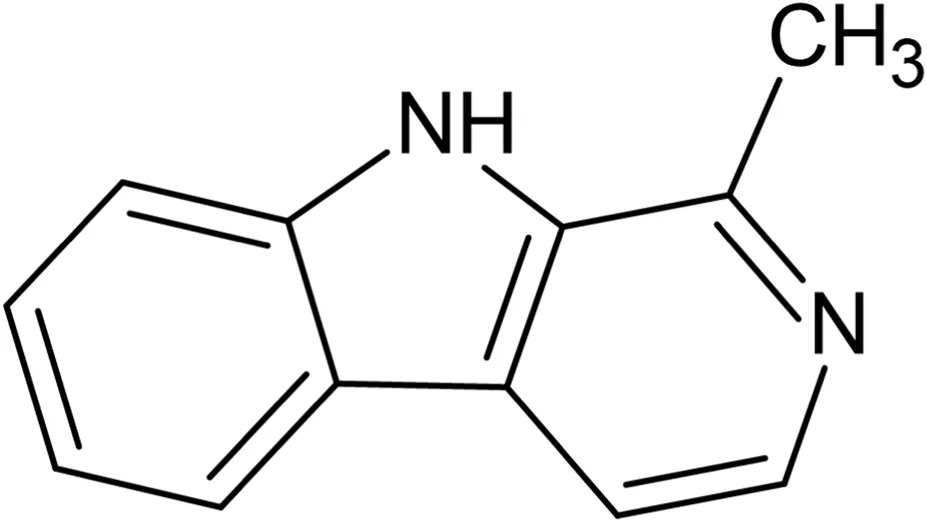
Chemical structure of HAR.

Harmane (HAR, Figure 1) is a beta-carboline, a naturally occurring, nonpolar heterocyclic aromatic amine (IUPAC name: 1-methyl-9H-pyrido [3,4-b]indole, CAS 486-84-0). It is found in many plant species; however, its presence and content may vary even within different parts of the same plant. Among others, significant amounts of HAR and similar beta-carboline alkaloids are typically found in *Banisteriopsis caapi* (commonly used in Ayahuasca) (Wang et al., 2010), *Peganum harmala* (Iranshahi et al., 2019), *Passiflora incarnata* (passionflower) (Aniszewski, 2007), *Passiflora coriacea* (Castolo-Sanchez et al., 2024), and *Tribulus terrestris* (Saeed et al., 2024), and it is a major component of many marketed food supplements worldwide. These products are typically marketed for their potential health benefits, including antidepressant/anxiolytic, neuroprotective, cognitive-enhancing, mood-regulating, and antioxidant properties. Beta-carbolines are also found in tobacco or tobacco smoke; their presence may contribute to its psychopharmacological effect (Aniszewski, 2007; Berlowitz et al., 2022; Hong et al., 2022). Several *Psilocybe* species also produce low amounts of β-carboline alkaloids, including HAR (Blei et al., 2020); accordingly, HAR has been hypothesized to exert an “entourage” effect on the hallucinogen psilocybin, possibly by interfering with psilocybin degradation (Blei et al., 2020). In addition to the natural sources, it is a product of the pyrolysis of proteins and amino acids during the heat-processing of the meat and coffee (de Meester, 1995; Herraiz, 2002; Pfau and Skog, 2004; Xie et al., 2021), and its presence in the human body has raised concerns regarding its physiological effects (Khan et al., 2017; Laviță et al., 2016; Louis et al., 2007).

### Effects of HAR on health and disease

HAR is linked to both harmful and beneficial effects on human health. With its derivatives harmine and norharman, it is the major component responsible for the antidepressant- and anxiolytic-like action of several herbal extracts (Berlowitz et al., 2022; Castolo-Sanchez et al., 2024; Ferraz et al., 2019). Antidepressant- and anxiolytic-like effects of HAR have been demonstrated in several animal models (Aricioglu et al., 2017; Ebrahimi-Ghiri et al., 2019; Farzin and Mansouri, 2006; Pandey et al., 2010). These effects have been attributed to the modulation of multiple neurotransmitter systems, including inhibition of monoamine oxidase (MAO) enzymes (Herraiz, 2023), serotonergic signaling (Abu Ghazaleh et al., 2015; Adell et al., 1996), and increased firing of dopaminergic neurons (Arib et al., 2010; Drucker et al., 1990). Further mechanisms relevant to these behavioral effects include inhibition of dopamine reuptake (Drucker et al., 1990) and inverse agonism at GABA_A receptors (Farzin et al., 2011).

A comprehensive review of HAR’s potential pharmacological activities was published by Khan et al. (2017). Except for the postulated neurotoxic/neuroprotective, anxiolytic, and antidepressant activities, for which HAR is perhaps best known, antidiabetic, antiplatelet, antioxidant, and anti-inflammatory activities have also been documented (Khan et al., 2017). On the contrary, beta-carbolines (including HAR) have also been investigated as neurotoxic agents (Sammi et al., 2018) and as possible contributors to essential tremor (Laviță et al., 2016; Quan et al., 2021).

### Pharmacokinetics and interaction potential of HAR

Beta-carbolines, including HAR, are substrates for cytochrome P450 (CYP) CYP1A1/2 and, to a minor extent, also for human CYP2D6, CYP2C19, and CYP2E1 (Herraiz et al., 2008). HAR has an oral bioavailability of 19.41% in rats and a distribution volume of 1.6 L/kg (Li et al., 2016). Its major metabolites are harmane-2-oxide, 6-hydroxy harmane, and 3-hydroxy harmane (Herraiz et al., 2008). Phase II metabolism, especially sulfation, predominated over phase I pathways. After intravenous administration, HAR showed rapid clearance (T_1/2_ of 4.71 h), while metabolites exhibited longer half-lives, indicating slower elimination and tissue accumulation (Li et al., 2016). Drug–drug interactions play a critical role in drug safety, with potential implications for the efficacy and toxicity of pharmaceutical compounds (Rendic, 2002). One of the key factors involved in drug metabolism and drug interactions is the CYP enzyme system, which is responsible for the biotransformation of a wide range of xenobiotics, including drugs (Ingelman-Sundberg, 2005). Understanding the influence of drug–drug and drug–herb interactions on CYP enzyme activity is vital for predicting and minimizing adverse effects in clinical practice. Zhao et al. (2011) reported that HAR noncompetitively inhibited CYP3A4 while having no effect on CYP2D6 metabolic activity in human liver microsomes (HLMs). To our knowledge, the *in vitro* study by Zhao et al. remains the only study specifically investigating the effects of harmane on cytochrome P450 activity, whereas data on its broader CYP-mediated interaction potential remain very limited. Thus, the aim of the proposed preclinical investigations was to evaluate the effects of HAR i) on CYP1A2, CYP2A1, CYP2B1, CYP2C6, CYP2C11, CYP2D1/2, and CYP3A1/2 enzymes in rats *in vivo,* ii) on the same rat CYP enzymes *in vitro,* and iii) on human CYP1A2, CYP2C9, CYP2D6, and CYP3A4 enzymes *in silico*.

## 2 Materials and methods

### 2.1 Study design

In this study, the experimental design should be considered exploratory. A reliable prospective sample size calculation was not feasible since both the expected effect size and the variance of the primary outcomes could not be estimated in advance. This limitation is common in preclinical/animal experiments addressing novel interventions, where prior data are insufficient to inform conventional power analyses. To assess the effects of HAR on selected hepatic CYP isoforms following subchronic *in vivo* administration, an 8-day intragastric dosing regimen was implemented in rats at doses of 25, 40, and 64 mg/kg/day. The dosing strategy was determined based on prior studies on the antidepressant-like effects of HAR by other researchers. Specifically, Farzin and Mansouri (2006), Aricioglu et al. (2017), and Pandey et al. (2010) demonstrated the antidepressant-like effects of intraperitoneally administered HAR in rats at doses of 5, 10, and 15 mg/kg, as well as 2.5, 5, and 10 mg/kg, respectively. Given the relatively low absolute bioavailability of harmane [approximately 19% (Guan et al., 2001; Li et al., 2016)], intragastric administration at 25, 40, and 64 mg/kg was selected to achieve systemic exposure comparable to the effective intraperitoneal doses reported in previous studies. Based on this *a priori* information and the 3R principles of animal welfare, the size of the rat group was also selected (n = 10/group).

### 2.2 Chemicals

HAR (purity 98%) and the following chemicals were purchased from Sigma-Aldrich (St. Louis, United States): 4′-hydroxydiclofenac, bovine serum albumin, acetic acid, chlorpropamide, dextromethorphan, dextrorphan, diclofenac, DL-laudanosine, EDTA, glucose-6-phosphate, glucose-6-phosphate dehydrogenase, ibuprofen, KCl, MgCl_2_ x 6 H_2_O, NADP, Na_2_HPO_4_, NH_3_, NH_4_HCO_3_, paracetamol, phenacetin, progesterone, sucrose, testosterone, and triethylamine. Propylene glycol was obtained from Fagron (Olomouc, CZE). The metabolites of testosterone (2β-, 2α-, 7α-, 6β-, 16α-, and 6β-hydroxytestosterone) were purchased from Steraloids Inc. (Newport, United States). Chemicals and organic solvents (methanol, acetonitrile, and isopropanol) used for HPLC analysis were purchased from Lach-ner (HPST, Prague, CZE) and were of an HPLC or LC-MS grade. Primary and secondary antibodies used for western blot analysis are listed in Supplementary Table S1.

### 2.3 Animals and treatments

Male Wistar albino rats (9 weeks old, weighing 403 ± 20 g) were obtained from the Masaryk University breeding facility (Brno, CZE) and were housed under standard laboratory conditions (temperature 22 °C ± 2 °C, humidity 55% ± 5%, and 12-h light/dark cycle), with unrestricted access to food and water. After 6 days of acclimatization, the rats were randomly divided into four groups of 10 animals. Animals were treated intragastrically with HAR suspended in a 75:25 mixture of propylene glycol and 5% glucose (*v/v*) (4 mL/kg) for 8 consecutive days. Propylene glycol was selected as the vehicle because suspension in water was not homogeneous, making precise dose measurement and administration through the intragastric probe impossible. In contrast, suspension in propylene glycol was homogeneous and smooth. The doses of HAR were 25, 40, and 64 mg/kg/day (groups H25, H40, and H64), and the control group (group C) received the corresponding volume of vehicle. The HAR doses were selected to encompass a wide range of previously observed biological activities *in vivo* (Aricioglu et al., 2017; Farzin and Mansouri, 2006).

The animals were sacrificed by decapitation 24 h after the last drug administration, following a fasting period of at least 12 h. The liver was isolated, sampled, and stored at −80 °C until microsomal fraction isolation. All experiments were carried out in an accredited animal facility (Accreditation No. 4546/2021-MZE-18134) in accordance with Czech Act No. 246/1992 and with the approval of both the local and the Czech Central Commission for Animal Welfare (MSMT-15858/2013-310).

### 2.4 Hematological and immunological parameters

To assess the possible toxic effects of HAR in rats, hematological and immunological parameters were analyzed at the Small Animal Clinic Laboratory, University of Veterinary Sciences Brno, CZE. On the last day of the experiment, 0.5 mL blood samples were collected into EDTA-containing test tubes, kept cold without direct contact with ice, and analyzed using a Sysmex XT-2000iV analyzer (Sysmex, JPN). White blood cell, platelet, and red blood cell count, hemoglobin and hematocrit levels, mean corpuscular volume (MCV), mean corpuscular hemoglobin (MCH), mean corpuscular hemoglobin concentration (MCHC), red blood cell distribution width (RDW), platelet distribution width (PDW), mean platelet volume (MPV), plateletcrit (PCT), and the white blood cell differential (segmented neutrophils, lymphocytes, monocytes, eosinophils, and basophils) were measured.

### 2.5 Malondialdehyde assay

Serum malondialdehyde levels were determined using a TBA assay coupled with HPLC. In brief, 50 µL serum was mixed with 1% H_3_PO_4_ and 0.43 M TBA, incubated at 95 °C for 60 min, and cooled on ice. MDA standards were prepared from 1,1,3,3-tetraethoxypropane. Fluorescence detection (527/551 nm) was performed by HPLC using an RP-18 column and methanol: phosphate buffer (3:2, v/v; pH 6.8) as the mobile phase at 0.8 mL/min.

### 2.6 Rat liver microsome preparation and determination of total protein and CYP content

The methods for liver collection and rat liver microsome (RLM) isolation by differential ultracentrifugation were described in our previous study (Dovrtelova et al., 2018). The total protein content in RLM was evaluated using a Pierce™ BCA Protein Assay Kit (Thermo Fisher Scientific, Waltham, United States) according to the manufacturer’s protocol and expressed as milligrams of protein per milliliter of RLM. Total and relative CYP and cytochrome P420 content were assessed by CO-difference spectroscopy according to the method of Omura and Sato (1964) and expressed as nanomoles of cytochrome P per milliliter of RLM.

### 2.7 CYP metabolic activity in the RLM of treated animals

The activities of CYP1A2, CYP2A, CYP2B, CYP2C6, CYP2C11, CYP3A1/2, and CYP2D1/2 were assessed by measuring the rate of production of CYP-specific metabolites by RLM with the NADPH-generating system according to a modified method of Wojcikowski et al. (2008). The rate of phenacetin O-deethylation (CYP1A2) (Dovrtelova et al., 2018), diclofenac 4′-hydroxylation (CYP2C6) (Noskova et al., 2016), testosterone hydroxylation (CYP2A1 (7α-), CYP2B (16β-), CYP2C11 (2α- and 16α-), CYP3A1/2 (6β-testosterone hydroxylation) (Dovrtelova et al., 2015), and dextromethorphan O-demethylation (CYP2D1/2) (Dovrtelova et al., 2018) were used as described previously. The details and minor modifications of the methods are summarized in Table 1. Both the incubation conditions and the analytical methods were internally validated, including Km determination, substrate saturation, product formation linearity (incubation) and analytical linearity, LOQ, LOD, precision, accuracy, and reproducibility. The final volume of incubation mixture was 0.5 mL, and it contained phosphate buffer (50 mM, pH 7.4), EDTA (1.1 mM), NADP (1.2 mM), glucose-6-phosphate (4.4 mM), MgCl_2_. 6 H_2_O (3.2 mM), glucose-6-phosphate-dehydrogenase (1.55 U in 1 mL), 50 µL of RLM (1 mg/mL of total protein content), internal standard, and a specific marker for determining the activity of CYP. The reaction was carried out at 37 °C in a horizontal vortex and stopped after incubation by adding 50 µL of ice-cold organic solvent (0 °C) and cooling on ice. Both the parent drug and CYP-specific metabolites formed during activity assays were measured using one of two HPLC systems: a Shimadzu LC-10 (Shimadzu, Tokyo, JPN) with a diode array detector (SPD-M10AVP) and fluorescence detector (RF10AXL) or a Dionex UltiMate 3000RS LC (Thermo Fisher Scientific, Waltham, United States) with a diode array detector. The effects of HAR premedication on CYP enzyme activities were evaluated within the linear range of product formation with respect to incubation time and probe substrate concentration. The metabolic activities of all CYP enzymes were expressed as the amount of metabolite formed (mol/min/mg total RLM protein) and are presented as a percentage of the reaction rate in the control group. All experiments were performed in triplicate.

**TABLE 1:** Cytochrome P450 metabolic activity—incubation and analytical conditions.

Cytochrome P450	1A2	2C6	2D1/2	2A1, 2B1, 2C11, and 3A1/2
Substrate	Phenacetin	Diclofenac	Dextromethorphan	Testosterone
Substrate concentration [μM]	400	100	500	400
Incubation time [min]	20	20	20	15
Metabolite	Acetaminophen	4′-Hydroxydiclofenac	Dextrorphan	7α-, 16β-, 2α-, 16α-, and 6β-testosterone
Internal standard	Chlorpropamide	Chlorpropamide	Laudanosine	Progesterone
HPLC system	Dionex UltiMate 3000RS LC	Dionex UltiMate 3000RS LC	Shimadzu LC-10	Dionex ultimate 3000RS LC
Column	Accucore PFP^1^	Accucore C18^1^	Tessek Phenyl^2^	Accucore C18^1^
Column oven [°C]	35	35	Room temperature (22 °C ±3 °C)	40
Mobile phase A	0.1% NH_4_CH_3_COO, pH 4.6	10 mM NH_4_HCO_3_, pH 7.1 (acetic acid)	20 mM KH_2_PO_4_ with 0.1% triethylamine, pH 3.8	1% methanol
Mobile phase B	Acetonitrile			
Flow [mL/min]	0.4	0.75	0.65	0.9
Gradient (A:B)	0 min 90:10 5 min 35:65	0 min 85:15	Isocratic flow 50:50	0 min 17:83
	5–7 min 35:65	4 min 79:21	​	10.5 min 19:81
	7–10 min 90:10	5.5 min 60:40	​	13.8 min 45:55
	​	5.7–6.5 min 50:50	​	14 min 60:40
	​	6.7–8 min 82:18	​	16 min 40:60
	​	​	​	16.2–19 min 15:85
Sample processing	Centrifugation, 4 min, 10,844 x g	Centrifugation, 4 min, 10,844 x g	L:L^3^ n-butanol:hexan 1:9	Centrifugation, 4 min, 10,844 x g
Detection	UV 245 nm	UV 276 nm	FLD λ ex 280 nm, λ em 320 nm	UV 254 nm

1 Accucore columns (100 × 3 mm, 2.6 µm, Thermo Fisher Scientific, United States) with pre-column.

2 Tessek column–Tessek phenyl (150 × 3 mm, 5 μm, Tessek, CZE).

3 L:L, liquid:liquid extraction + evaporation under the gentle N_2_ stream; FLD, fluorescence detection.

### 2.8 Western blotting

To measure the content of CYP isoforms with significantly altered metabolic activity (CYP2B, CYP2C11, CYP2D1/2, CYP3A1, and CYP3A2), the western blot method was performed as described previously (Dovrtelova et al., 2018). Each sample was analyzed in triplicate. Diluted microsomal samples of control animals were pooled (no statistically significant differences in the amount of CYP between individual control samples were found). β-actin was used as the loading control. The transfer quality was visually evaluated after total protein staining with Ponceau S. The specifications and dilutions of the primary and secondary antibodies used are listed in Supplementary Table S1.

### 2.9 Inhibitory assays of HAR with RLM

To study the effect of HAR on CYP isoforms (CYP1A2, CYP2A, CYP2B, CYP2C6, CYP2C11, CYP3A1/2, and CYP2D1/2) in pooled drug-naïve RLM, inhibitory assays were performed with slightly modified methods described in Section 2.7 (the reaction conditions are described in Table 2). The final concentration of HAR in the samples of the pilot experiment were 100, 10, 1, and 0.1 µM. The concentration of RLM was 0.25 mg/mL in the sample, with a total volume of 1 mL. Control samples were incubated with an equivalent volume (20 µL) of the vehicle used for the HAR-treated samples (20% methanol in 10 mM HCl). The reaction conditions were within the linear range, and the substrate concentrations were adjusted to Km values, as determined in our previous experiments. Since HAR inhibited CYP2A1 and CYP3A1/2 in pilot incubations, further experiments were performed to obtain IC_50_ and Ki values. The concentrations of HAR in the samples were 150, 100, 50, 10, 7, 3.5, 1, 0.75, 0.5, and 0.1 µM, whereas the concentration of testosterone was 100 µM for IC_50_ evaluation. In the determination of the Ki, the concentrations of HAR were 100, 75, 50, and 10 µM, whereas those of testosterone were 50, 100, and 200 µM. The IC_50_ evaluation was performed using GraphPad Prism software (version 10.4.0 (621); GraphPad Software, Boston, United States), and Ki was calculated using SigmaPlot (version 15.0.0.13, Grafiti, Palo Alto, United States). The experiments were performed in triplicate. The inhibitor potency was evaluated as follows: an IC_50_ value < 1 µM suggests strong inhibition, an IC_50_ value of 1–10 µM indicates moderate inhibitory potency, and an IC_50_ value > 10 µM suggests weak inhibitory potency and may have no clinical impact (Jin et al., 2015; Wang et al., 2021).

**TABLE 2:** Specific conditions of RLM inhibitory assays.

Substrate	Concentration in the incubation mixture [µM]	Duration of incubation [min]	RLM protein concentration [mg/mL]	CYP isoform	Metabolite quantified
Phenacetin	210	40	0.25	CYP1A2	Paracetamol
Diclofenac	8	30		CYP2C6	4′-Hydroxydiclofenac
Dextromethorphan	28	40		CYP2D1/2	Dextrorphan
Testosterone	100	20		CYP3A1/2	6β-Hydroxytestosterone

### 2.10 *In silico* modeling

#### 2.10.1 Protein preparations

Crystal structures of all studied proteins were downloaded from the RCSB PDB (Berman et al., 2000). Only human protein structures were used for further *in silico* analyses as rat CYP structures were not available. The protein structures selected for individual cytochrome targets, with their respective PDB IDs and mapping to rat orthologs, are shown in Table 3. Waters, ions, and ligands were cleaned from the PDB files, except for heme groups. The H++ webserver (Gordon et al., 2005), with the default settings, was used to add hydrogen atoms to the selected apoprotein structures. Heme-group parametrizations were carried out using a Python-based Metal Center Parameter Builder (MCPB.py) (Li and Merz, 2016). Protein hydrogen atoms were optimized to avoid any clashes using AMBER (Case et al., 2005) software with the ff14SB (Maier et al., 2015) force field in the IGB7 (Mongan et al., 2007) implicit solvent model environment.

**TABLE 3:** Selected PDB structures of human CYP proteins and their closest rat orthologs with available crystal structure.

Rat	Human	PDB code
CYP1A2	CYP1A2	2HI4
CYP2C11	CYP2C9	1R9O
CYP2D1/2	CYP2D6	3TBG
CYP3A1	CYP3A4/5	1TQN

#### 2.10.2 Ligand preparation

Ligands were downloaded from ChemSpider (Pence and Williams, 2010) in SMILES format. Three-dimensional (3D) geometry generation of compounds was carried out using the LigPrep (Schrödinger, 2018) module with the Optimized Potentials for Liquid Simulations (OPLS3e) force field (Harder et al., 2016). Protonation and tautomerization states were calculated using Epik (Shelley et al., 2007) implemented in LigPrep. Ligands with a state-penalty value from 0 to 1 were selected for further docking calculation and scoring steps. Prepared ligand geometries were optimized using MOPAC2016 (Stewart, 2016) software at the PM6-D3H4X6 (Brahmkshatriya et al., n.d.) level with the implicit solvent model COSMO (Klamt and Schüürmann, 1993) and parametrized using the Antechamber (Wang et al., 2006; Wang et al., 2004) module implemented in AMBER software with the partial atomic charges calculated at the level of DFTB3 (Gaus et al., 2011).

#### 2.10.3 Molecular docking

PLANTS docking software (Korb et al., 2006) was used for pose generation. The docking center was approximately 2.3 Å above the iron atom of the heme group in the binding pocket. Docking radii were set to 15 Å, 15 Å, 10 Å, and 15 Å for 1R9O, 1TQN, 2HI4, and 3TBG, respectively, to include the entire inner catalytic cavity and the access tunnels. We generated at least 100 poses per ligand and increased the number by 10 for each rotatable bond in the ligand structure.

#### 2.10.4 Geometry optimizations and clustering

Protein–ligand complex geometries were prepared using tLEaP implemented in AMBER software. Only the ligand atoms and hydrogen atoms of the protein within 4 Å of the ligand poses were allowed to move. An initial molecular mechanics optimization step was applied with the ff14SB force field *in vacuo* to quickly eliminate clashes and form complementary interactions. The optimization step was followed by clustering of the conformations to reduce the final number by eliminating conformationally similar complexes. Another step of molecular mechanics optimization was also applied, together with IGB7 solvation, to calculate the score by accounting for desolvation upon interactions. The Cuby4 computational chemistry framework was used for the optimization and clustering calculations (Řezáč, 2016).

#### 2.10.5 Scoring

In this study, the affinity of ligands for proteins was estimated as the sum of the protein–ligand interaction energy in implicit solvent and the conformational free energy change in the ligand and protein (only hydrogens for proteins) upon complexation. Complexes with ligand strain energy penalties greater than 5 kcal/mol were discarded during scoring.

### 2.11 Statistical analyses

After evaluation using histograms and Shapiro–Wilk test, most data were considered non-normally distributed. The non-parametric Kruskal–Wallis test, followed by multiple comparisons of mean ranks for all groups, was used for analyses (total CYP content, total protein content, and metabolic activities). The results from the western blot were evaluated using the Wilcoxon signed-rank test followed by Bonferroni correction. Statistica software (version 14.0.0.15, TIBCO Software Inc., Palo Alto, United States) was used. All values are presented as the median and interquartile range. The results were considered statistically significant when *p* ≤ 0.05.

## 3 Results

### 3.1 Effects of HAR on rats

#### 3.1.1 Animal status and hematological and immunological parameters

The weight gain in experimental animal groups was negatively correlated with the HAR dose during the experiment (Supplementary Table S2). Four animals died prematurely in the group administered with the highest dose of HAR. However, an autopsy did not reveal the cause. There was no significant difference in liver weight between animal groups (data not shown).

The increase in the white blood cell count, MCHC, platelet count, PDW, MPV, PCT (Supplementary Table S3), and the white blood cell differential (segmented neutrophils, monocytes, eosinophils, and basophils) were observed (Supplementary Table S4). No effects of HAR administration were found in the red blood cell count, hemoglobin, hematocrit, MCV, MCH, and RDW (Supplementary Table S3).

#### 3.1.2 Total protein, cytochrome P450, and cytochrome P420 content

**FIGURE 2:**
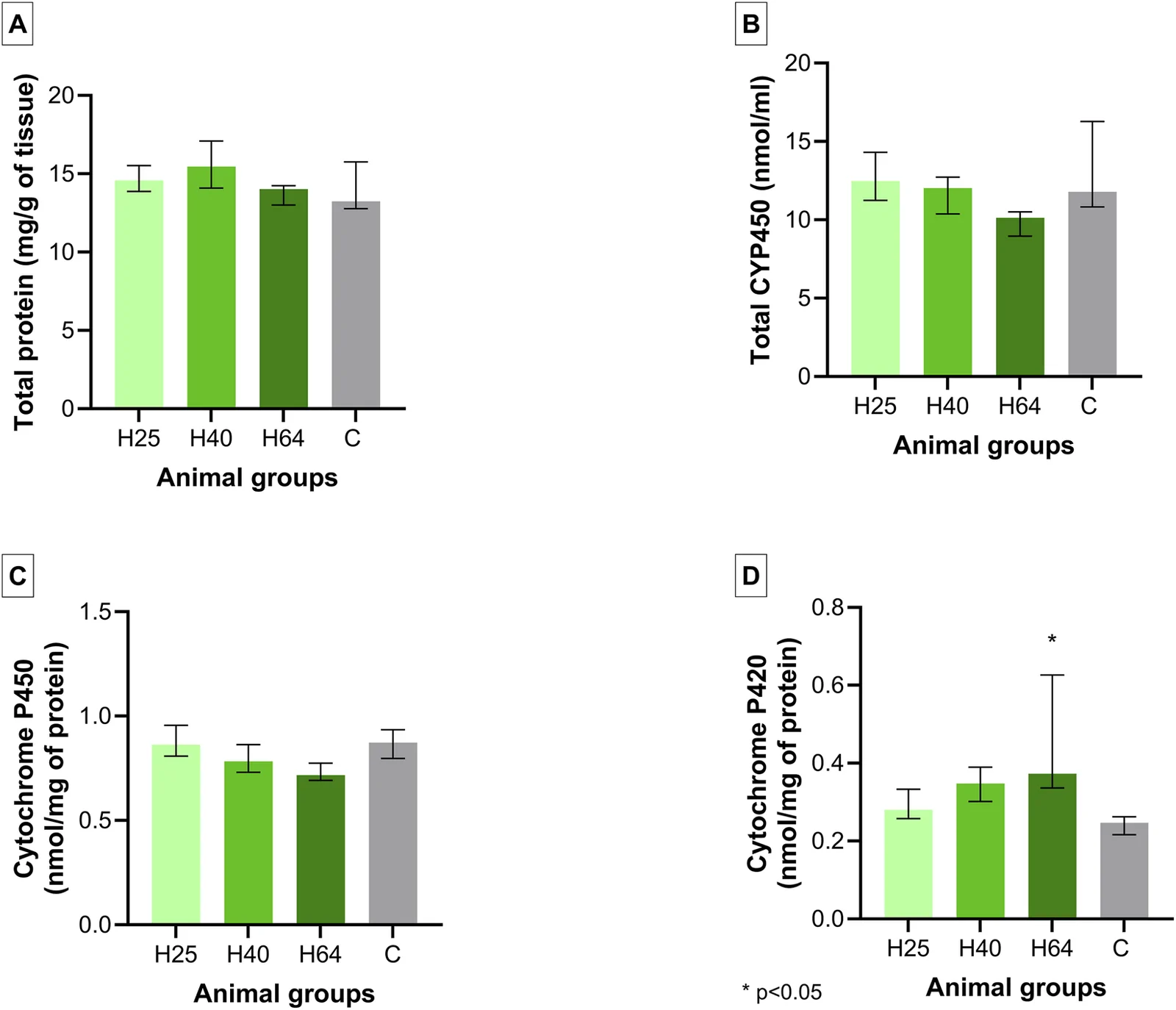
Total protein content **(A)**, total CYP content **(B)**, relative CYP content **(C)**, and relative cytochrome P420 content **(D)** in RLM of animals treated with 25 mg/kg/day (H25 group, N = 10), 40 mg/kg/day (H40, N = 10), and 64 mg/kg/day (H64, N = 6) of HAR. Columns represent medians, and error bars represent the interquartile range. *p < 0.05 compared to the control (N = 9).

The total protein content was not affected by the administration of HAR at doses of 25, 40, and 64 mg/kg/day compared with the control group (*p* = 0.287) (Figure 2A). Similarly, the relative content of CYP (nmol CYP per 1 mg of total protein content, Figure 2C) and the absolute content of CYP (nmol/ml of RLM), as shown in Figure 2B, do not significantly differ (*p* = 0.060 and *p* = 0.206). However, the relative content of the inactive form of CYP, cytochrome P420, was significantly increased in the H64 group compared to that in the control group (*p* = 0.014, Figure 2D).

#### 3.1.3 CYP metabolic activity in the RLM of treated animals

**FIGURE 3:**
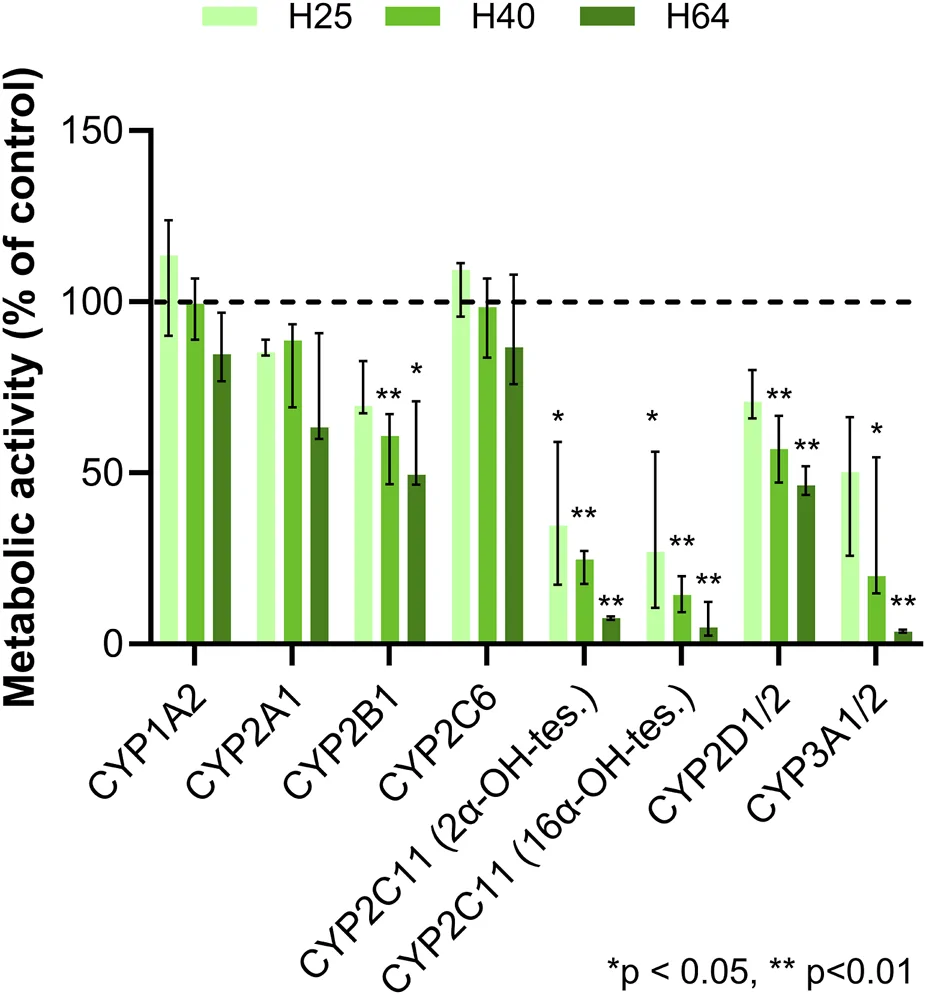
Metabolic activities of CYP1A2, CYP2A1, CYP2B1, CYP2C6, CYP2C11, CYP2D1/2, and CYP3A1/2 after systemic administration of HAR at doses of 25 mg/kg/day (H25 group, N = 10), 40 mg/kg/day (H40, N = 10), and 64 mg/kg/day (H64, N = 6). Data are presented as the median of the percentage of the control and the interquartile range. *p < 0.05 and **p < 0.01 compared to the control (N = 9).

Premedication with HAR decreased the metabolic activity of all tested CYP enzymes. In particular, the metabolic activity of CYP1A2, CYP2A1, and CYP2C6 decreased modestly and dose-dependently, with maximum reductions to 84.6%, 63.3%, and 86.7% of the control median, respectively (Figure 3). A strong inhibitory effect on CYP2B1, CYP2C11, CYP2D1/2, and CYP3A1/2 was also observed with the highest dose of HAR. The metabolic activity was decreased to 49.4% [CYP2B1 (16β-OH testosterone)], 7.5% [CYP2C11 (2α-OH testosterone)], 4.9% [CYP2C11 (16α-OH testosterone)], 46.3% [CYP2D1/2 (dextrorphan)], and 3.6% [CYP3A1/2 (6β-OH testosterone)] of the median of the control. The result data are summarized in Table 4.

**TABLE 4:** Metabolic activity in pmol/min/mg of the protein content for CYP1A2, CYP2A1, CYP2B1, CYP2C6, CYP2C11 (represented by 16α- and 2α-OH-testosterone), CYP2D1/2, and CYP3A1/2.

​	Dose of HAR (mg/kg/day)	N	Mean	SD	Median	LQ	UQ	*p*
CYP1A2	25	10	1,515.31	243.62	1,556.11	1,232.91	1,695.88	​
	40	10	1,293.17	314.46	1,361.84	1,218.28	1,462.21	​
	64	6	1,170.21	175.95	1,159.30	1,052.65	1,326.33	​
	C	9	1,468.56	247.70	1,369.72	1,277.61	1,612.36	​
CYP2A1	25	10	226.47	31.48	223.35	220.79	233.21	​
	40	10	215.93	50.91	232.56	181.17	245.02	​
	64	6	185.79	56.51	165.79	157.06	238.00	​
	C	9	272.99	68.09	262.08	229.11	275.68	​
CYP2B1	25	10	39.70	5.06	37.55	36.38	44.59	​
	40	10	32.18	7.27	32.82	25.17	36.28	**
	64	6	30.03	7.19	26.65	25.15	38.29	*
	C	9	52.97	8.59	53.96	50.93	60.02	​
CYP2C6	25	10	405.93	41.78	426.22	373.06	433.91	​
	40	10	353.05	112.78	384.13	326.60	416.47	​
	64	6	362.12	88.31	338.27	296.05	420.88	​
	C	9	462.99	124.64	390.01	382.16	503.74	​
CYP2C11 (2α-OH-testosterone)	25	10	709.96	501.54	598.27	155.97	830.82	*
	40	10	446.73	270.39	427.98	137.79	292.51	**
	64	6	184.79	138.21	130.12	36.48	183.05	**
	C	9	1804.56	535.22	1732.28	1,216.90	1736.72	​
CYP2C11 (16α-OH-testosterone)	25	10	511.91	443.08	398.97	155.97	830.82	*
	40	10	275.08	239.76	212.30	137.79	292.51	**
	64	6	109.77	106.52	72.10	36.48	183.05	**
	C	9	1,566.87	600.18	1,479.53	1,216.90	1736.72	​
CYP2D1/2	25	10	907.18	162.48	882.63	821.50	997.75	​
	40	10	717.53	181.39	709.50	587.00	830.00	**
	64	6	606.17	100.17	576.38	542.75	647.50	**
	C	9	1,283.47	163.04	1,245.50	1,172.25	1,352.50	​
CYP3A1/2	25	10	285.20	165.93	291.49	149.64	384.90	​
	40	10	189.14	151.82	115.43	86.33	316.91	*
	64	6	32.00	27.34	20.87	19.57	24.61	**
	C	9	573.03	171.23	580.80	571.11	677.98	​

*p*, statistical significance (*, *p* < 0.05; **, *p* < 0.01 versus the control group).

#### 3.1.4 Western blotting

**FIGURE 4:**
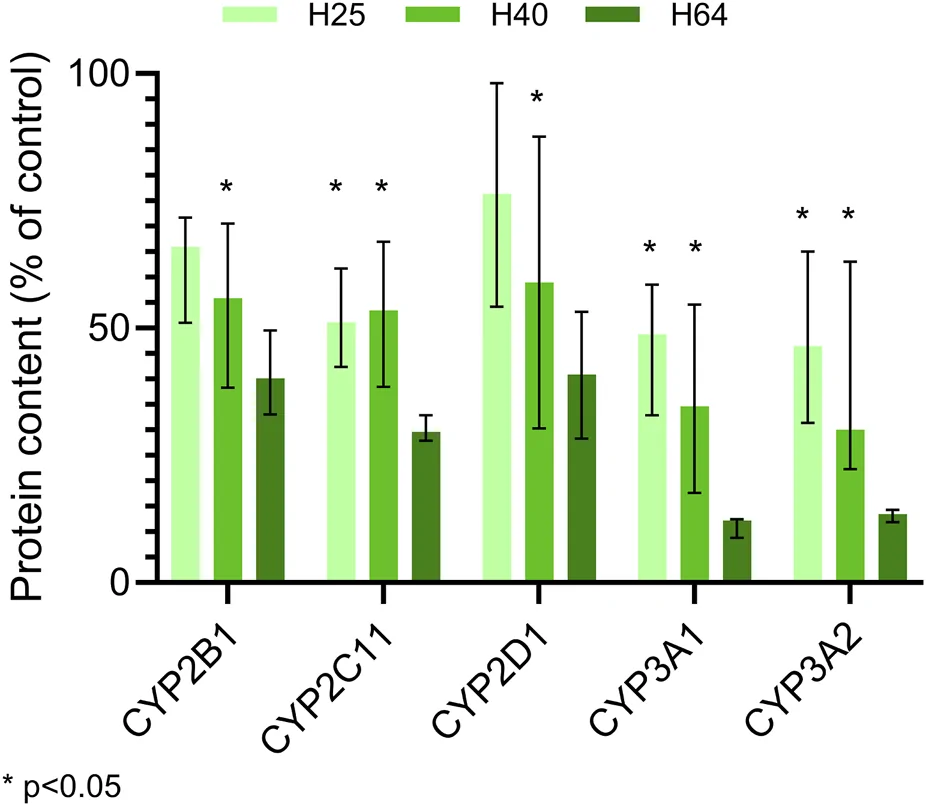
CYP2B1, CYP2C11, CYP3A1, CYP3A2, and CYP2D1 content in RLM of animals treated with HAR [25 mg/kg (H25 group, N = 10), 40 mg/kg/day (H40, N = 10), and 64 mg/kg/day (H64, N = 6)] expressed as a percentage of the control. Columns represent medians, and error bars represent the interquartile range. *p < 0.05 compared to the control (N = 9).

A significant dose-dependent decrease in the content of CYP2B1, CYP2C11, CYP2D1, CYP3A1, and CYP3A2 in RLM of HAR-treated animals was observed (Figure 4). The decrease in CYP content compared with the controls can also be observed on representative western blot membranes (Supplementary Figures S1–S5 in the Supplementary data). Although the changes were not significant in the H64 group, probably due to the low number of animals (N = 6), they were clearly distinguishable.

### 3.2 *In vitro* inhibitory assays

**FIGURE 5:**
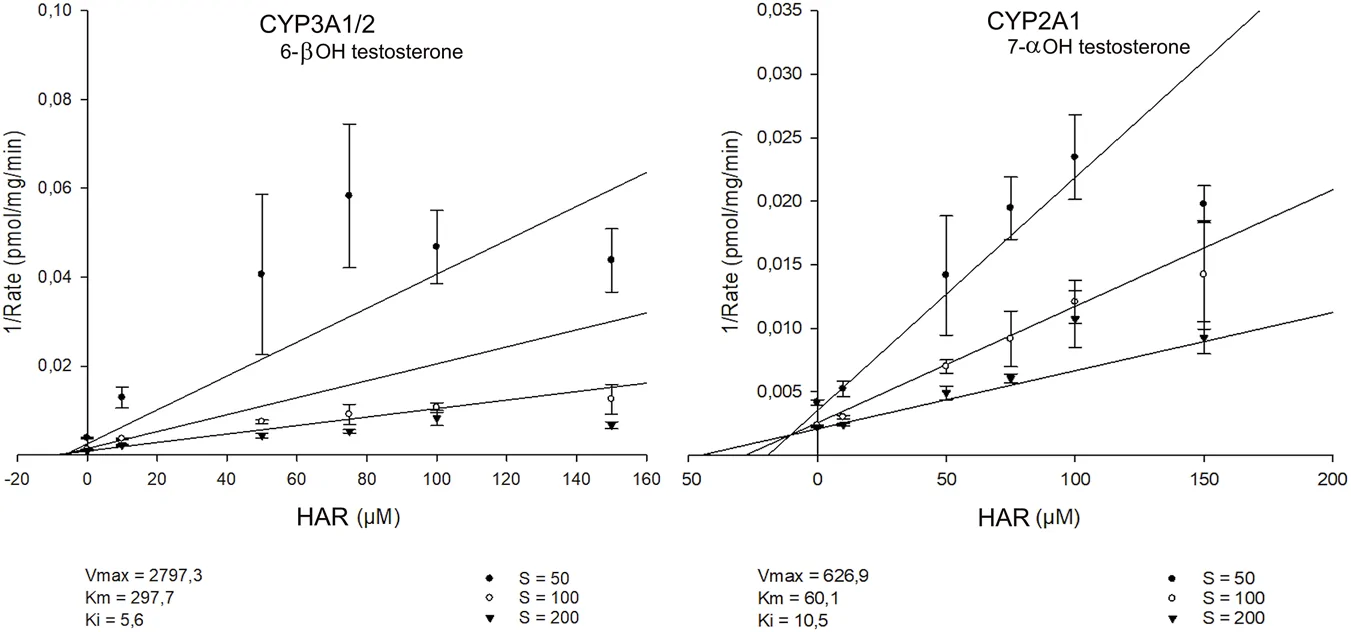
Dixon plots showing the reciprocal of the reaction rate (1/ν) versus increasing initial concentrations of HAR for testosterone 6β-hydroxylation (CYP3A4) and testosterone 7α-hydroxylation (CYP2A1). The experiments were performed in triplicate.

Inhibitory assays conducted in drug-naïve RLM demonstrated that HAR is a moderate noncompetitive inhibitor of rat CYP3A1/2 and a moderate mixed-type inhibitor of CYP2A1. For CYP3A1/2, the inhibition constant (Ki) was determined to be 5.6 µM (95% CI: 3.4–7.4), with an IC_50_ value of 1.2 µM (95% CI: 0.92–1.42 µM). In the case of CYP2A1, the Ki was 10.4 µM (95% CI: 7.0–13.9), and the IC_50_ value was 14.0 µM (95% CI: 9.4–20.9 µM). The inhibition type was determined based on visual inspection of Dixon plots, the sum of squared residuals, Akaike information criterion (AIC) values, standard errors, and 95% confidence intervals (Figure 5). No inhibition was observed for CYP1A2, CYP2B, CYP2C6, CYP2C11, or CYP2D2 in pilot experiments (data not shown).

### 3.3 *In silico* modeling of the interaction between HAR and rat and human CYP enzymes

**FIGURE 6:**
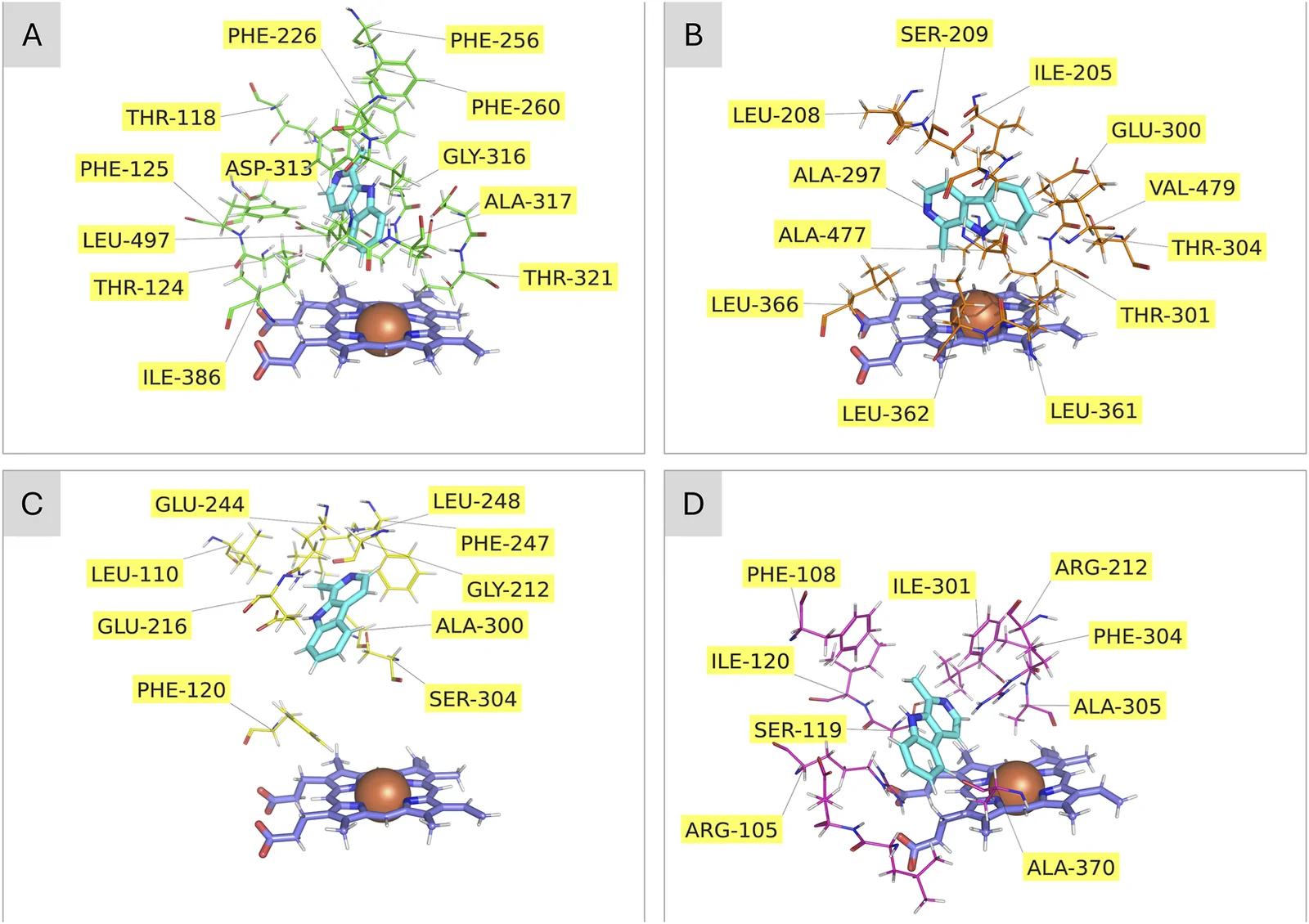
Interactions of HAR (light blue) with the active site residues of CYP1A2 **(A)**, CYP2C9 **(B)**, CYP2D6 **(C)**, and CYP3A4 **(D)**. Heme groups are colored in purple.

**FIGURE 7:**
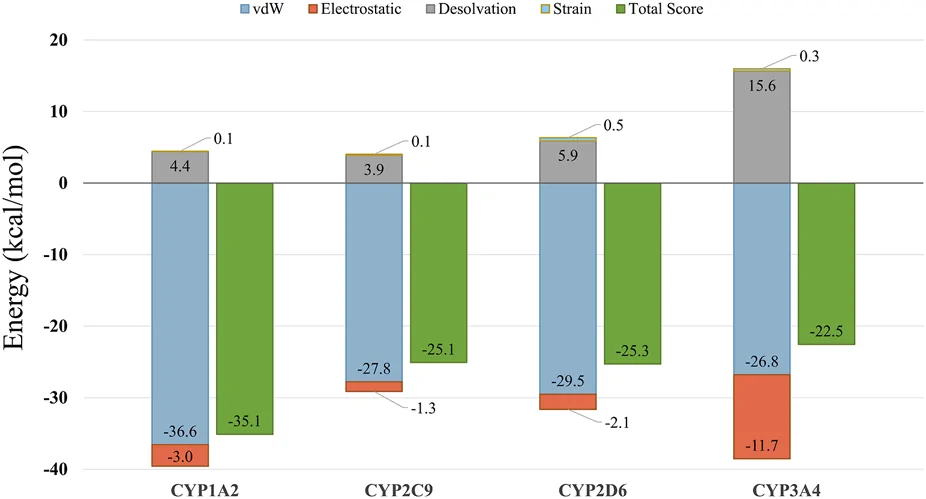
HAR complexes with CYP1A2, CYP2C9, CYP2D6, and CYP3A4 proteins: contributions of electrostatic and van der Waals (vdW) interactions (attractive) and desolvation penalty (repulsive) terms to the total scores.

HAR was docked into all four human CYP enzymes. During the analysis, we mainly focused on the positioning of HAR in the active site, the interpretation of the interactions between HAR and different residues, and the comparison of binding modes among different CYP enzymes. HAR was preferably located close to the heme group of the proteins (4.3–5.7 Å from the iron atom). Only in the case of CYP2D6 was HAR found to be further away, in the access tunnel. This was due to the reorganization of residues caused by the binding of the native ligand in the protein crystal structure. As a result, PHE-120 was pushed closer to the heme group, creating steric hindrance preventing HAR from moving closer to the heme (Figure 6). HAR is a nonpolar aromatic molecule that primarily interacts with aliphatic hydrophobic and aromatic amino acids. Therefore, the main contribution to the binding energy comes from van der Waals forces, whereas electrostatic interactions are rather less significant (Figure 7). The hydrophobic nature of HAR also contributes to a low desolvation penalty. The molecule’s small size and planar structure lead to a negligible strain energy penalty of only up to 0.5 kcal/mol due to ligand binding.

As shown in Figure 7, electrostatic interactions are markedly increased in CYP3A4. In CYP3A4, the primary contribution to the electrostatic interaction arises from a hydrogen bond with SER-199, followed by contributions from ARG-212 and the iron ion (Figure 6D). However, these interactions also result in additional desolvation penalties (15.6 kcal/mol), compensating for the gain from electrostatic interactions in the total score. The majority of interactions in CYP1A2, CYP2C9, and CYP2D6 occur with nonpolar amino acid residues. Particularly, the stacking interactions by PHE residues (226, 256, and 260) in CYP1A2 provide stronger stabilization of the HAR molecule (Figure 6A).

## 4 Discussion

As mentioned in Section 2.1, the doses used in our *in vivo* study were selected based on the antidepressant-like effect and 19% oral bioavailability described by other authors (Aricioglu et al., 2017; Farzin and Mansouri, 2006; Li et al., 2016). However, during the experiment, a decrease in relative weight was observed, with a trend to correlate with the dose (Supplementary Table S2), and four animals in the H64 group died prematurely. The autopsy was performed for the gross examination of all major organs and body cavities to assess abnormalities, such as lesions, tumors, inflammation, hemorrhage, and organ size or morphology, but did not reveal any pathological changes. From the hematological parameters analyzed (Supplementary Tables S3, S4) increases were observed in white blood cell count, MCHC, platelet count, and PCT in the experimental groups, particularly in the H40 and H64 groups, compared with those in the controls. Additionally, elevated levels of segmented neutrophils, monocytes, and eosinophils were detected. These hematological alterations may indicate a systemic response to the administered substance as they represent nonspecific biomarkers associated with various pathological conditions, including inflammation, hematological dysregulation, or even early-stage malignancy. The observed leukocytosis, along with increased platelet parameters, suggests a potential pro-inflammatory or stress-related response, which could be indicative of subclinical toxicity. Furthermore, the concentration of malondialdehyde was insignificantly increased in the H64 group, indicating a mild enhancement of oxidative stress potentially connected with inflammatory processes (Supporting Information Supplementary Figure S6). Chronic inflammation and immune dysregulation are implicated in the pathogenesis of various diseases, including cancer and hematological disorders (Grivennikov et al., 2010). Further investigation is warranted to determine whether the observed hematological changes are transient adaptive responses or reflect a narrow margin between antidepressant-like and toxic doses of HAR. Hagiwara et al. (1992a) performed an experiment focused on the renal tubular toxicity of HAR in F344 rats. Animals were administered HAR at the doses of 41 and 81 mg/kg/day in the diet for 28 days. In line with our results, the weight of animals treated with 81 mg/kg/day HAR was significantly lower than that of the control, but no significant differences in liver weights were observed (Hagiwara et al., 1992a). In addition, 74 mg/kg/day HAR administered orally in the diet for 6 weeks to F344 rats did not exhibit liver toxicity. A reduction in body weight was described (Hagiwara et al., 1992b). Wang et al. (2023) studied the effect of 8-week administration of HAR (0.1 mg/kg) in a rat diabetic model. Significantly increased ALT and decreased urea levels indicated potential hepatic and renal toxicity of HAR, even though histopathology did not reveal any changes. Moreover, moderate muscle fiber and axonal degeneration were observed peripherally, but no significant changes were revealed after pathological examinations of rat brains. Increased levels of superoxide dismutase and TNF-α indicated oxidative stress resulting from HAR treatment (Wang et al., 2023). Even though the maximal doses and the duration of HAR administration mentioned exceed those used in our experiment, it is possible that HAR exhibited nonspecific toxicity at a dose of 64 mg/kg/day. The LD_50_ value of intraperitoneally administered HAR in mice is 66 ± 20 mg/kg (Dolzhenko et al., 1982). Considering the bioavailability of 19%, the relative exposure to harmane in our study was lower, approximately 12 mg/kg at the highest dose.

After an 8-day administration of HAR, a significant decrease in metabolic activity per protein content was observed in CYP2B1, CYP2C11, CYP2D1/2, and CYP3A1/2 compared to the control group. In accordance with the metabolic activity evaluation, the western blot analysis revealed that the contents of the CYP2B1, CYP2C11, CYP2D1, CYP3A1, and CYP3A2 proteins were also reduced. Therefore, HAR reduced the metabolic activity of the abovementioned isoforms probably via their downregulation. General characterization of RLMs showed a slight, non-significant decrease in both the relative and absolute content of CYP with increasing doses of HAR. This trend was accompanied by a significant decrease in the protein content of selected isoforms as determined by western blot. Notably, the relative increase in cytochrome P420 content observed in the H64 group may be associated with the toxic effects of HAR. Dose-dependent models were not applied as the range of doses was only exploratory, and the activity–dose relationship was investigated using the full probabilistic Bayesian Hill model, which relies on rather strong assumptions (Supplementary Material: Hill model S1).

The factors related to the reduction in CYP protein levels, which might be affected by HAR intake, include coactivators, corepressors, specific transcription factors, and post-translational modifications of nuclear receptors (e.g., phosphorylation, SUMOylation, ubiquitination, or acetylation) (Kumar and Perdew, 2018; Pavek, 2016; Smutny et al., 2013). Moreover, gene expression may be regulated by histone modification or DNA methylation (leading to changes in chromatin formation or the prevention of transcriptional factor binding) (Ghotbi et al., 2009; Tamási and Falus, 2012). Posttranscriptional mechanisms (miRNA and chemical modifications of mRNA) may also play a role. Furthermore, HAR might influence CYP content indirectly by altering hormone levels or hormonal regulation, resulting in the modulation of enzyme expression. Since sex-specific differences may occur, it would be necessary to confirm the results with female rats (Kato and Yamazoe, 1992).

Interestingly, as indicated by the hematological findings (Supplementary Tables S3, S4), the administration of HAR might cause immune changes in rats. The impact of inflammatory processes on CYP expression and metabolic activity has been extensively documented. Several studies have reported cytokine-mediated suppression of CYP isoform transcription (Aitken et al., 2006; de Jong et al., 2020). In addition, oxidative stress and inflammation-associated hepatic nitric oxide production may further contribute to these effects (Zanger and Schwab, 2013). However, further experiments are required to determine whether these mechanisms were involved in the presented findings.

Previous studies have demonstrated that HAR induced CYP1A1 mRNA in HepG2 (El Gendy and El-Kadi, 2010), H4IIE (El Gendy and El-Kadi, 2010; Hümmerich et al., 2004), and MCF-7 (Hümmerich et al., 2004) cells in a time- and concentration-dependent manner and also induced the CYP1A1 protein level, probably via the aryl hydrocarbon receptor. In line with our findings, Hümmerich et al. (2004) reported no significant increase in CYP1A1 activity, as measured by the ethoxyresorufin-O-deethylase assay. El Gendy and El-Kadi (2010) observed an increase in CYP1A1 enzyme activity after the co-incubation of HepG2 cells with HAR. However, this effect was modest, even at the highest concentration, compared with a 1 nM model inducer. Although these findings are partially inconsistent with our results, no studies to date have evaluated the effects of HAR on CYP enzymes *in vivo*, and *in vitro* models cannot fully replicate the complexity of a whole organism. Moreover, HAR has been shown to modulate the hypothalamo-pituitary–adrenal axis (Smith et al., 2013), which may further influence CYP expression.

The *in vitro* inhibitory effect of HAR on CYP3A1/2 was demonstrated with IC_50_ values ranging from 1.17 to 1.22 µM. In accordance with our findings, Zhao et al. (2011) reported that HAR acts as a noncompetitive inhibitor of CYP3A4 (IC_50_ = 0.73 ± 1.06 µM, Ki = 1.66 μM) and had no inhibitory effect on CYP2D6 after *in vitro* incubation of HAR with HLMs. For context, multiple studies have reported the IC_50_ value of the potent CYP3A1/2 inhibitor ketoconazole ranging from 0.20 to 0.78 μM (Eagling et al., 1998; Rioux et al., 2013; Turan et al., 2001), with a Ki value of 0.132 μM (Wang et al., 2022) in RLM assays. These findings confirm HAR’s significant inhibitory potency against CYP3A enzymes in rats.

The *in silico* modeling suggests that HAR primarily interacts with CYP enzymes through van der Waals forces due to its hydrophobic and aromatic nature, with electrostatic interactions playing a minor role, except in CYP3A4. Although HAR binds near the heme group in most CYPs, steric hindrance in CYP2D6 prevents close positioning, potentially reducing its inhibitory effect. Given its favorable binding in CYP1A2, CYP2C9, and CYP2D6, particularly through stabilizing stacking interactions, HAR may act as an inhibitor of these enzymes, although its impact on CYP3A4 remains less certain due to a compensatory desolvation penalty.

HAR and norharman are present in various foodstuffs, with levels significantly influenced by processing conditions. Major sources of human exposure include coffee, seasonings, cooked meats, and alcoholic beverages. Given the relative content of HAR in food and beverages in the range of tens to hundreds of ng/g or µg/L, with the exception of roasted sesame seeds, in which the content may increase to units of mg/kg due to roasting (Liu et al., 2022). This assumes that intake is tens to hundreds of micrograms daily (Herraiz, 2004). Cigarette smoke may be a significant contributor to the HAR exposure as well because smokers had several-fold higher concentrations of HAR in urine than nonsmokers (Jain, 2018). However, the concentrations of HAR found in the urine of the smokers were several orders lower than concentrations theoretically achieved in our settings [doses up to 64 mg/kg, bioavailability of 19%, and Vd of 1.9 L/kg (Li et al., 2016) may predict peak concentrations of HAR ≥10 mg/L].

Although the hematological findings did not reveal any kind of toxicity even at the dose of 64 mg/kg and other authors have not reported toxicity with HAR at similar dosages (Hagiwara et al., 1992a; Hagiwara et al., 1992b), the situation may have been different in our study. We assessed the drug-metabolizing potential of HAR at higher doses (up to 64 mg/kg), which may exert cytotoxic effects in the *in vitro* test system. However, cytotoxicity was not evaluated in the present study as it was beyond the primary scope of this investigation. Although the general prevalence of using food supplements is very high in Western countries (Rozga et al., 2013), the absence of robust demographic information on harmane supplementation represents a limitation of the clinical interpretation of our study*.* Currently, there is no universally recommended dosage of HAR as a food supplement. It varies depending on the specific product. Thus, given the wide range of contents found in food supplements, HAR may be of relevant clinical importance regarding its interactions with the low therapeutic index drugs metabolized by the human ortholog CYP. The examples of these drugs are presented in Supplementary Table S5.

## 5 Conclusion

In conclusion, the present study demonstrated that an 8-day administration of HAR led to a dose-dependent reduction in the metabolic activity and protein expression of rat liver CYP enzymes, including CYP2B1, CYP2C11, CYP2D1/2, and CYP3A1/2, likely through downregulation. The observed trends indicate a biologically relevant effect, especially when considering that ranked tests and false-positivity correction were applied. The modulation of CYP expression may be attributed to multiple regulatory pathways, including transcriptional and post-transcriptional mechanisms, as well as hormonal or inflammatory influences. Additionally, *in vitro* data suggest that HAR inhibited CYP2A1 and CYP3A1/2. *In silico* modeling also revealed interactions with human CYP enzymes; HAR interacts with CYP enzymes predominantly via van der Waals forces, exhibiting noncompetitive inhibition of CYP3A4. Although this study was conducted in an animal model, the observed dose-dependent downregulation of key CYP isoforms suggests that HAR, a promising herbal antidepressant, could significantly alter drug metabolism if similar effects occur in humans. Given the essential role of these enzymes in the biotransformation of many therapeutics, HAR-induced modulation may alter drug efficacy, increase the risk of adverse effects, or cause potential drug interactions. The intensity of these effects, particularly the substantial reduction in CYP2B1, CYP2C11, and CYP3A1/2 activity, indicates a potentially clinically relevant impact, especially for drugs with a narrow therapeutic index. However, caution is warranted as interspecies differences in CYP regulation necessitate further clinical validation before drawing definitive conclusions.

## Data Availability

The original contributions presented in the study are included in the article/Supplementary Material; further inquiries can be directed to the corresponding author.
