# Supplementary material for: Harmane inhibits hepatic cytochrome P450 enzymes in rats
Source: Front Pharmacol. 2026 Aug 17;17:1863858. doi: 10.3389/fphar.2026.1863858 (PMC13526679; doi:10.3389/fphar.2026.1863858)
Supplement: Supplementary file 1 [file Supplementaryfile1.zip › Supplementary tables.DOCX]

# Table S1: Primary and secondary antibodies used in the western blot analysis

| Antibody | Supplier, Cat. No. | Biological source | Species | Dilution |
| --- | --- | --- | --- | --- |
| Anti-CYP2B1/2 | Santa Cruz  Biotechnology  (sc-73546) | Mouse | Mouse, rat | 1:1000 |
| Anti-CYP2C11 | Cypex (R-PAP 121) | Goat |  | 1:  5 000 |
| Anti-CYP2D1 | LifeSpan  BioSciences  (LS-C22614-100) | Rabbit | Rat | 1:  4 000 |
| Anti-CYP3A1 | Santa Cruz  Biotechnology  (sc-53246) | Mouse | Mouse, rat, human | 1:2000 |
| Anti-CYP3A2 | Cypex (R-PAP 171) | Rabbit | Rat | 1:5 000 |
| Anti-  β-actin | Cell Signalling  Technology  (cs-4970) | Rabbit | Mouse, rat, human, monkey,  bovine, pig | 1:2000 |
| Anti-mouse | Sigma-Aldrich (A4416) | Goat |  | 1:5000 |
| Anti-goat | Sigma-Aldrich (A5420) | Rabbit |  | 1:5000 |
| Anti-  rabbit | Sigma-Aldrich (A0545) | Goat |  | 1:5000 |

**Table S2:** Relative weight of animals premedicated with HAR.

| Dose of HAR (mg/kg/day) | Mean relative weightn (%) | SD (%) |
| --- | --- | --- |
| 25 | 0.36 | 1.94 |
| 40 | -3.69 | 4.57 |
| 64 | -7.38 | 3.32 |
| C | 2.86 | 1.67 |

**Table S3:** Haematological parameters of rats subchronically administered with HAR. LQ - lower quartile, UQ - upper quartile

| Dose of HAR (mg/kg/day) | N | Mean | | SD | Median | LQ | UQ |  |
| --- | --- | --- | --- | --- | --- | --- | --- | --- |
| White blood cell count (x10^9/l) | | | | | | | | |
| 25 | 8 | 17.38 | | 3.11 | 16.62 | 15.17 | 19.12 |  |
| 40 | 10 | 20.66 | | 5.85 | 20.54 | 18.51 | 23.55 |  |
| 64 | 4 | 24.94 | | 6.07 | 26.75 | 20.41 | 29.47 |  |
| C | 7 | 11.73 | | 2.92 | 10.99 | 10.33 | 14.27 |  |
| MCHC (g/l) | | | | | | | | |
| 25 | 8 | 361.38 | | 7.29 | 361.50 | 355.00 | 365.00 |  |
| 40 | 10 | 365.60 | | 9.22 | 364.00 | 358.00 | 369.00 |  |
| 64 | 4 | 371.75 | | 6.50 | 373.00 | 367.00 | 376.50 |  |
| C | 7 | 354.86 | | 6.52 | 357.00 | 348.00 | 360.00 |  |
| Platelet count (x10^9/l) | | | | | | | | |
| 25 | | 8 | 1295.25 | 95.08 | 1283.50 | 1225.50 | 1344.50 |  |
| 40 | | 10 | 1416.20 | 179.27 | 1362.00 | 1339.00 | 1489.00 |  |
| 64 | | 4 | 1519.50 | 106.78 | 1488.50 | 1439.50 | 1599.50 |  |
| C | | 7 | 1022.71 | 290.63 | 1135.00 | 970.00 | 1177.00 |  |
| PDW (fl) | | | | | | | | |
| 25 | | 8 | 8.84 | 0.34 | 8.85 | 8.65 | 9.15 |  |
| 40 | | 10 | 8.53 | 0.38 | 8.45 | 8.30 | 8.90 |  |
| 64 | | 4 | 7.90 | 0.58 | 8.00 | 7.55 | 8.25 |  |
| C | | 7 | 8.81 | 0.29 | 8.80 | 8.60 | 8.90 |  |
| MPV (fl) | | | | | | | | |
| 25 | | 8 | 7.80 | 0.21 | 7.80 | 7.70 | 8.00 |  |
| 40 | | 10 | 7.49 | 0.28 | 7.50 | 7.40 | 7.70 |  |
| 64 | | 4 | 7.08 | 0.34 | 7.15 | 6.85 | 7.30 |  |
| C | | 7 | 7.70 | 0.24 | 7.70 | 7.40 | 7.80 |  |
| PCT (l/l) | | | | | | | | |
| 25 | | 8 | 0.010 | 0.001 | 0.010 | 0.010 | 0.010 |  |
| 40 | | 10 | 0.011 | 0.001 | 0.010 | 0.010 | 0.011 |  |
| 64 | | 4 | 0.011 | 0.001 | 0.011 | 0.010 | 0.012 |  |
| C | | 7 | 0.008 | 0.002 | 0.009 | 0.008 | 0.009 |  |
| Red blood cell count (10^12/l) | | | | | | | | |
| 25 | | 8 | 9.02 | 0.32 | 9.01 | 8.81 | 9.18 |  |
| 40 | | 10 | 9.65 | 0.77 | 9.60 | 9.32 | 9.99 |  |
| 64 | | 4 | 9.29 | 0.13 | 9.33 | 9.20 | 9.37 |  |
| C | | 7 | 9.11 | 0.51 | 9.01 | 8.80 | 9.38 |  |
| Haemoglobin (g/l) | | | | | | | | |
| 25 | | 8 | 162.50 | 5.21 | 162.50 | 157.50 | 167.00 |  |
| 40 | | 10 | 172.60 | 12.94 | 174.00 | 165.00 | 182.00 |  |
| 64 | | 4 | 168.00 | 3.83 | 167.00 | 165.00 | 171.00 |  |
| C | | 7 | 164.71 | 8.94 | 162.00 | 158.00 | 172.00 |  |
| Haematocrit (l/l) | | | | | | | | |
| 25 | | 8 | 0.45 | 0.01 | 0.45 | 0.44 | 0.46 |  |
| 40 | | 10 | 0.47 | 0.03 | 0.48 | 0.45 | 0.49 |  |
| 64 | | 4 | 0.45 | 0.02 | 0.45 | 0.44 | 0.46 |  |
| C | | 7 | 0.47 | 0.02 | 0.46 | 0.45 | 0.49 |  |
| MCV (fl) | | | | | | | | |
| 25 | | 8 | 46.18 | 11.04 | 50.60 | 47.10 | 51.25 |  |
| 40 | | 10 | 48.99 | 1.72 | 49.05 | 48.10 | 50.10 |  |
| 64 | | 4 | 48.70 | 1.47 | 48.05 | 47.90 | 49.50 |  |
| C | | 7 | 50.94 | 0.80 | 50.70 | 50.20 | 51.60 |  |
| MCH (pg) | | | | | | | | |
| 25 | | 8 | 18.03 | 0.49 | 17.90 | 17.70 | 18.35 |  |
| 40 | | 10 | 17.89 | 0.50 | 17.80 | 17.50 | 18.20 |  |
| 64 | | 4 | 18.10 | 0.34 | 18.10 | 17.85 | 18.35 |  |
| C | | 7 | 18.07 | 0.33 | 18.00 | 17.90 | 18.40 |  |
| RDW (%) | | | | | | | | |
| 25 | | 8 | 17.88 | 1.07 | 17.95 | 17.20 | 18.15 |  |
| 40 | | 10 | 18.78 | 1.13 | 18.55 | 18.40 | 18.80 |  |
| 64 | | 4 | 18.10 | 0.37 | 18.15 | 17.85 | 18.35 |  |
| C | 7 | 18.17 | | 1.33 | 18.50 | 17.50 | 19.20 |  |

**Table S4:** White blood cell differential of rats subchronically administered with HAR. LQ - lower quartile, UQ - upper quartile

| Dose of HAR  (mg/kg/day) | N | Mean | SD | Median | LQ | UQ |  |
| --- | --- | --- | --- | --- | --- | --- | --- |
|  |  |  |  |  |  |  |  |
| Segmented neutrophils (x10^9/l) | | |  |  |  |  |  |
| 25 | 8 | 6.89 | 1.40 | 7.29 | 5.72 | 8.08 |  |
| 40 | 10 | 8.92 | 4.74 | 7.78 | 5.91 | 9.32 |  |
| 64 | 4 | 10.47 | 1.87 | 10.46 | 8.86 | 12.09 |  |
| C | 7 | 1.33 | 0.21 | 1.32 | 1.13 | 1.49 |  |
| Lymphocytes (x10^9/l) | | |  |  |  |  |  |
| 25 | 8 | 8.85 | 2.54 | 8.65 | 6.72 | 10.61 |  |
| 40 | 10 | 9.77 | 2.88 | 10.10 | 9.18 | 12.23 |  |
| 64 | 4 | 11.87 | 4.64 | 12.01 | 8.32 | 15.43 |  |
| C | 7 | 9.82 | 2.88 | 9.58 | 7.92 | 12.40 |  |
| Monocytes (x10^9/l) | |  |  |  |  |  |  |
| 25 | 8 | 1.00 | 0.29 | 0.93 | 0.80 | 1.19 |  |
| 40 | 10 | 1.20 | 0.35 | 1.26 | 0.99 | 1.37 |  |
| 64 | 4 | 1.30 | 0.66 | 1.39 | 0.76 | 1.84 |  |
| C | 7 | 0.43 | 0.14 | 0.41 | 0.33 | 0.57 |  |
| Eosinophils (x10^9/l) | |  |  |  |  |  |  |
| 25 | 8 | 0.618 | 0.309 | 0.555 | 0.340 | 0.920 |  |
| 40 | 9 | 0.807 | 0.500 | 0.770 | 0.570 | 1.060 |  |
| 64 | 4 | 1.200 | 0.672 | 1.195 | 0.620 | 1.780 |  |
| C | 7 | 0.171 | 0.057 | 0.160 | 0.120 | 0.240 |  |
| Basophils (x10^9/l) | |  |  |  |  |  |  |
| 25 | 8 | 0.023 | 0.007 | 0.020 | 0.020 | 0.030 |  |
| 40 | 9 | 0.052 | 0.029 | 0.060 | 0.030 | 0.070 |  |
| 64 | 4 | 0.085 | 0.061 | 0.065 | 0.040 | 0.130 |  |
| C | 7 | 0.027 | 0.013 | 0.020 | 0.020 | 0.040 |  |

**Table S5:** Drugs metabolised via CYP3A4, CYP2D6 and CYP2C19

| CYP3A4 | | CYP2D6 | | CYP2C19 |
| --- | --- | --- | --- | --- |
| Immunosuppressants | Cyclosporine | **CNS drugs** | Nortriptyline | Phenytoin |
|  | Tacrolimus |  | Desipramine | Voriconazole |
|  | Sirolimus |  | Amitriptyline | Clopidogrel |
| Anticoagulants | Warfarin^1^ |  | Imipramine | Pazopanib |
|  | Rivaroxaban |  | Venlafaxine | Palbociclib |
|  | Apixaban |  | Haloperidol | Cabozantinib |
| Antiretrovirals | Saquinavir |  | Risperidone |  |
|  | Atazanavir |  | Aripiprazole |  |
|  | Darunavir | **Cardiovascular drugs** | Flecainide |  |
| Anticancer Agents | Vincristine |  | Propafenone |  |
|  | Vinblastine | **Opioids^2^** | Codeine |  |
|  | Etoposide |  | Tramadol |  |
|  | Imatinib |  |  |  |
|  | Erlotinib |  |  |  |
|  | sunitinib |  |  |  |
| Cardiovascular drugs | Amiodarone |  |  |  |
|  | Quinidine |  |  |  |
|  | Dronedarone |  |  |  |
| CNS drugs | Alprazolam |  |  |  |
|  | Carbamazepine |  |  |  |
|  | Diazepam |  |  |  |
| Opioids^2^ | Fentanyl |  |  |  |
|  | Alfentanil |  |  |  |
|  | Methadone |  |  |  |

^1^ Primarily CYP2C9 substrate, but also CYP3A4 to some extent.

^2^ While not all opioids undergo therapeutical drug monitoring, the change in the rate of metabolism affects conversion to active metabolites (e.g., morphine from codeine) which may lead to significant clinical consequences.
