# Supplementary material for: Harmane inhibits hepatic cytochrome P450 enzymes in rats
Source: Front Pharmacol. 2026 Aug 17;17:1863858. doi: 10.3389/fphar.2026.1863858 (PMC13526679; doi:10.3389/fphar.2026.1863858)
Supplement: Supplementary file 1 [file Supplementaryfile1.zip › Probabilistic Bayesian Hill model.PDF]

Supplementary material

Table of contents

Hill model with CYP activity between zero and one 1
Hill model with relaxed minimal CYP activity 3

Hill model with CYP activity between zero and one

The probabilistic version of the Hill model was used to model the dose-dependent curves of individual CYP isoforms.

$$
\begin{aligned}
Activity_i &\sim \text{Student}\ (\mu_i, \sigma, \nu) \\
\mu_i &= \text{Max} - \frac{\text{Max} - \text{Min}}{1 + \left(\frac{\log\left(Concentration_{CYP[i]}\right)}{IC_{50[CYP]}}\right)^{-Hill_{CYP}}} \\
IC_{50[j]} &\sim \text{HalfNormal(1, 2)} \quad \text{, for j = 1..9} \\
Hill_j &\sim \text{HalfNormal}\ (5, 1) \quad \text{, for j = 1..9} \\
\text{Max} &\sim \text{Constant}\ (1) \\
\text{Min} &\sim \text{Constant}\ (0) \\
\sigma &\sim \text{Exponential}\ (1) \\
\nu &\sim \text{Gamma(2, 0.1)}
\end{aligned}
$$

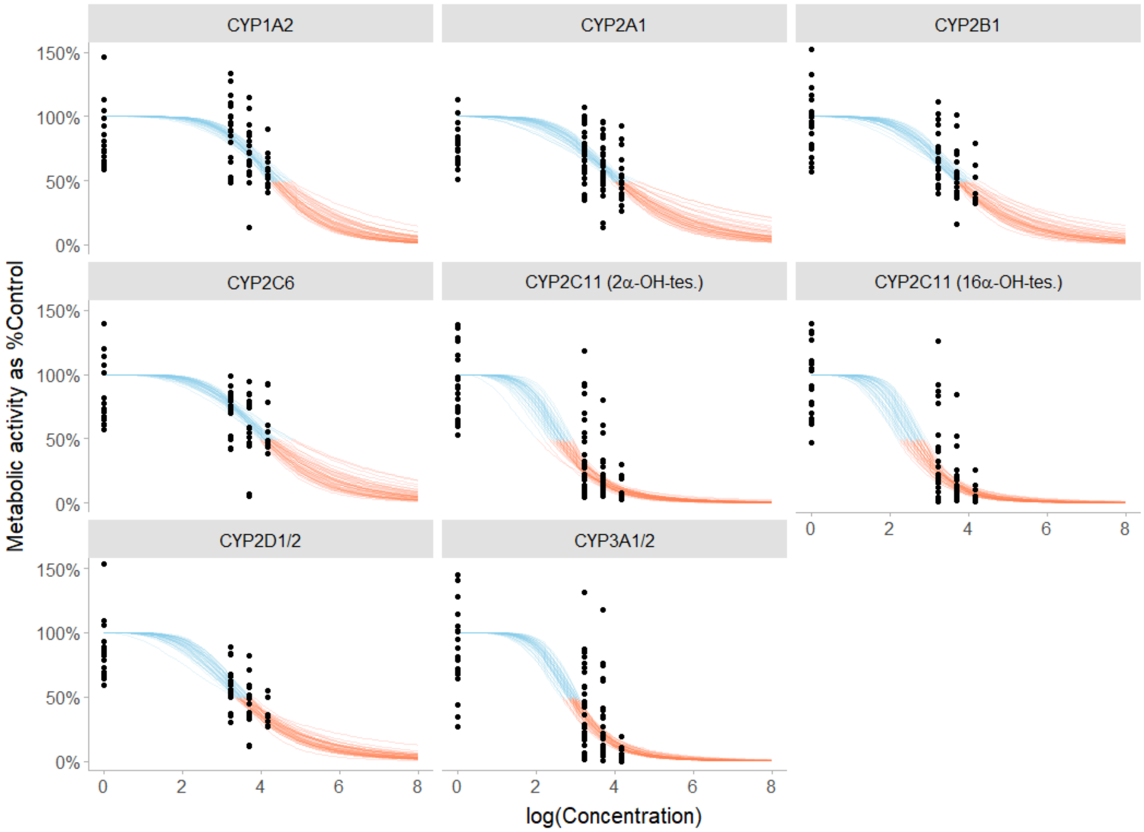

Figure 1: A spaghetti plot of plausible sigmoid curves from the Hill model. The blue and red colors represent metabolic activity being higher or lower than 50 %, respectively.

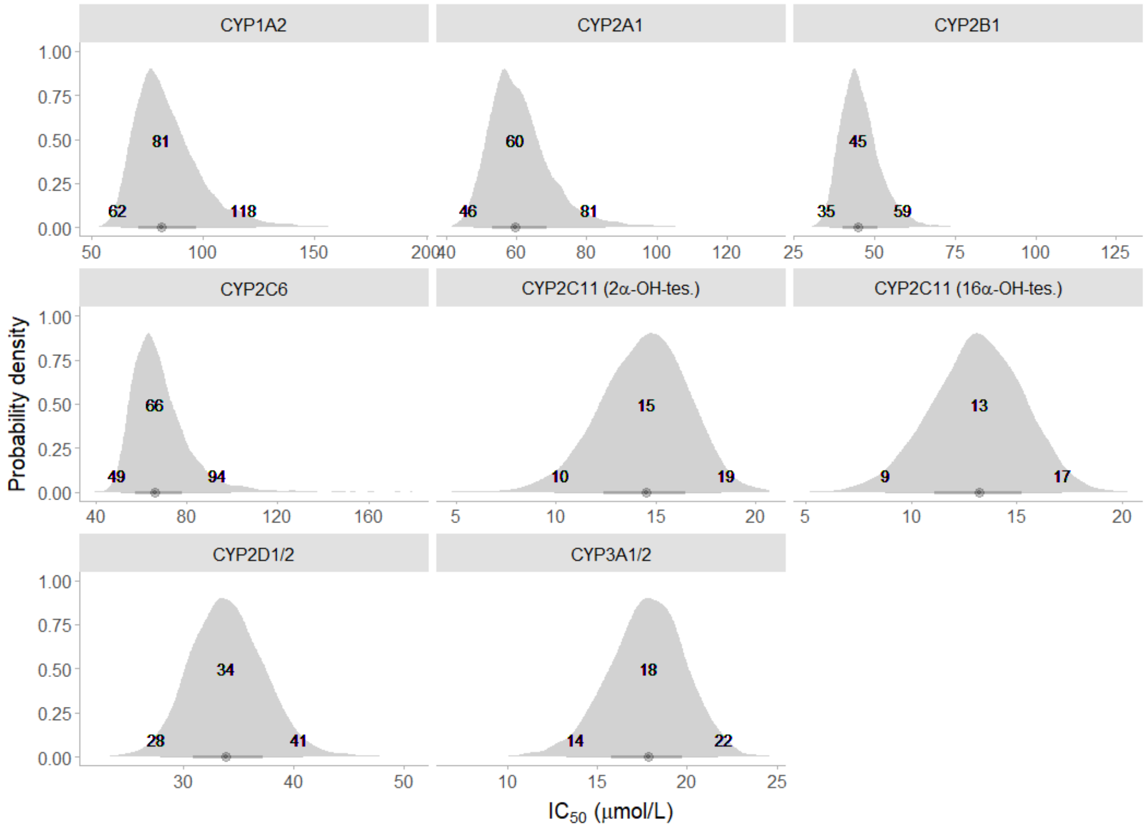

Figure 2: Probabilistic distributions of IC_{50} parameters obtained from the Hill's equation. The numbers represent 0.05, 0.5, and 0.95 quantiles of the distributions.

Hill model with relaxed minimal CYP activity

IC_{50} values were determined from the Hill model below with slightly modified priors to capture the greater variability in effective inhibition of specific isoforms. A_{min} stands for minimal achieved activity in data.

$$
\begin{aligned}
Activity_i &\sim \text{Student }(\mu_i, \sigma, \nu) \\
\mu_i &= \text{Max} - \frac{\text{Max} - \text{Min}}{1 + \left(\frac{\log\left(Concentration_{CYP[i]}\right)}{IC_{50[CYP]}}\right)^{-Hill_{CYP}}} \\
IC_{50[j]} &\sim \text{HalfNormal}(5,\ 1) \quad \text{, for j} = 1..9 \\
Hill_j &\sim \text{HalfNormal }(5, 1) \quad \text{, for j} = 1..9 \\
\text{Max} &\sim \text{Normal }(1, 0.1) \\
\text{Min} &\sim \text{Exponential }\left(1/\text{A}_{min[CYP]}\right) \\
\sigma &\sim \text{Exponential }(1) \\
\nu &\sim \text{Gamma}(2,\ 0.1)
\end{aligned}
$$

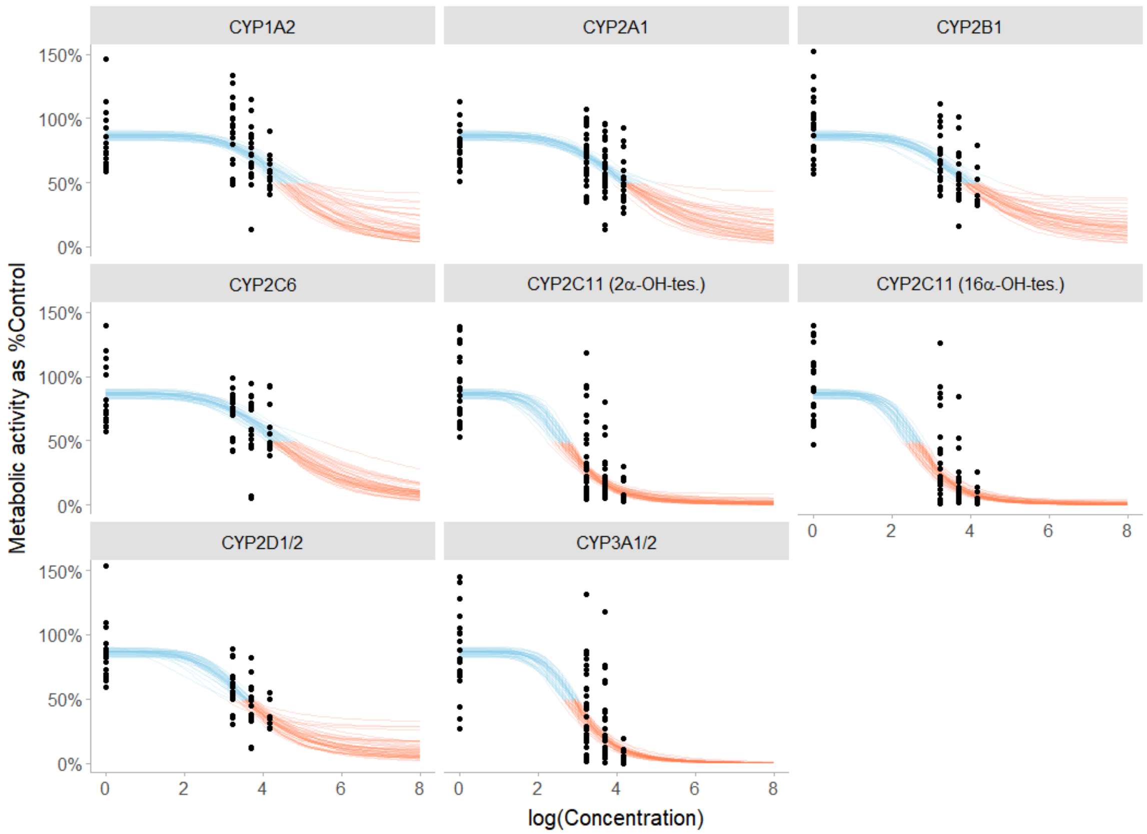

Figure 3: A spaghetti plot of plausible sigmoid curves from the Hill model. The blue and red colors represent metabolic activity being higher or lower than 50 %, respectively.

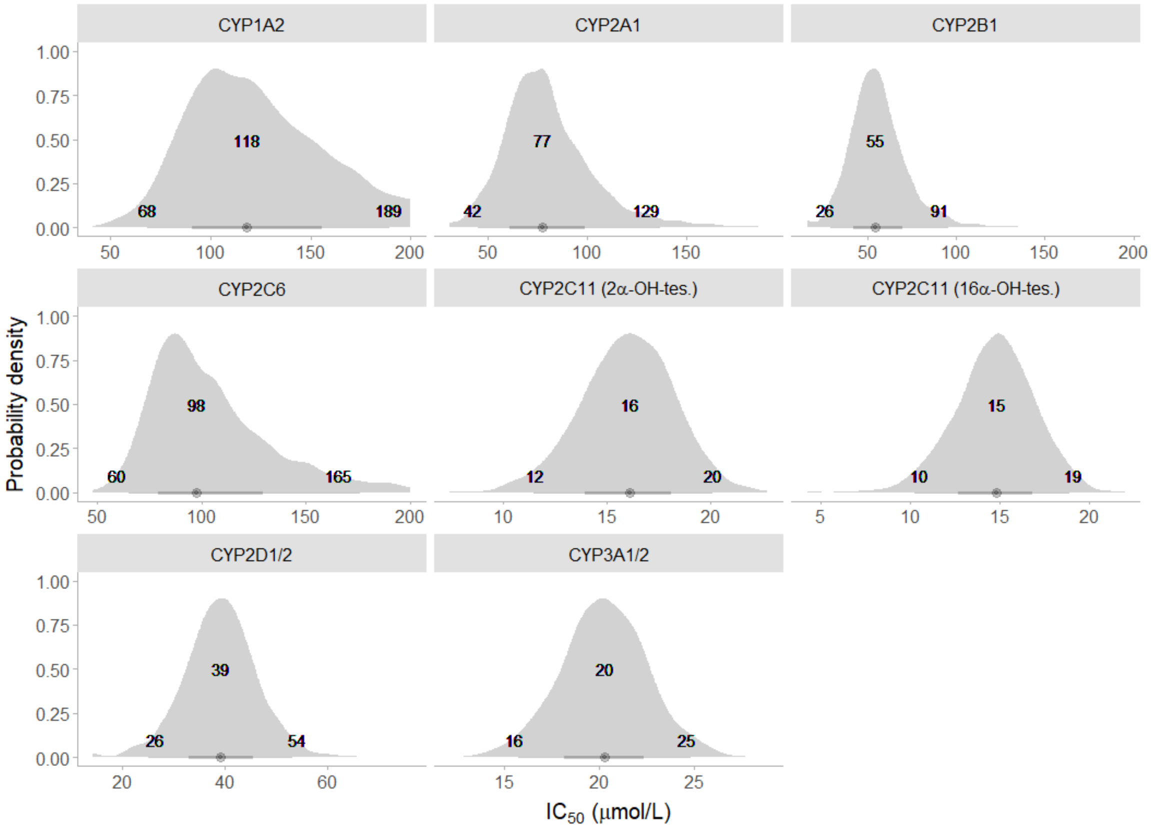

Figure 4: Probabilistic distributions of IC_{50} parameters obtained from the Hill's equation. The numbers represent 0.05, 0.5, and 0.95 quantiles of the distributions.
